# Driven to stay or leave: Exploring motivation, engagement, and turnover intentions among pharmacists in the healthcare system

**DOI:** 10.1016/j.rcsop.2025.100645

**Published:** 2025-08-22

**Authors:** Muna Sabah Murad, Mohammad Waheedi, Fatima Jeragh Alhaddad, Maryam Yousef Baqer, Farah Atallah Alenezi

**Affiliations:** a Faculty of Pharmacy, Kuwait University, Kuwait; b Open Space Consulting, Kuwait; c Ministry of Health, Kuwait

**Keywords:** Motivation, Self-determination theory, Engagement, Turnover intentions, Pharmacists, Structural equation modeling

## Abstract

**Background:**

High pharmacist turnover remains a global concern, as pharmacists' intention to leave their jobs can lead to increased financial burdens and reduced quality of pharmaceutical care. Understanding the psychological and organizational factors that drive this intention to leave is essential for designing effective retention strategies.

**Objectives:**

This study aimed to identify the variables related to motivational needs and work engagement which are associated with pharmacists' intention to leave.

**Methods:**

A cross-sectional study was conducted using a self-administered questionnaire among 288 pharmacists in eight hospitals. The survey assessed multidimensional work motivation, job engagement (UWES-9), and turnover intention. Structural equation modeling (SEM) was used to analyze the interrelations between constructs and demographic variables.

**Results:**

Work engagement was negatively associated with turnover intention (β = −0.49), Amotivation was directly associated with higher turnover intention (β = 0.17) and lower engagement (β = − 0.10). Intrinsic motivation had a statistically significant and positive effect on work engagement (β = 0.81). Pharmacists in public hospitals reported higher turnover intention than those in private hospitals (β = − 0.19). Pharmacists less than 40 years old and those in certain hospitals exhibited higher amotivation and intention to leave. Organizational setting influenced several motivational types, with intrinsic, extrinsic social motivation and identified motivation more prevalent among public sector pharmacists.

**Conclusion:**

Work engagement and motivation were critical factors impacting pharmacists' turnover intention, with organizational context and age acting as important moderators. Strategies aimed at boosting intrinsic motivation and work engagement, particularly for younger pharmacists, are vital for decreasing turnover and fostering a more stable pharmacy workforce within healthcare systems.

## 1 Introduction

Employee turnover presents a significant challenge for many organizations, as it not only results in the loss of skilled personnel but also incurs substantial costs associated with recruitment and replacement.^1^ A study conducted in New Mexico in 2004 reported that annual turnover costs for healthcare providers ranged from $17 to $29 million, representing approximately 3.4 % to 5.8 % of the institution's $500 million annual operating budget.^1^ Pharmacists, as highly accessible healthcare professionals, play a critical role in delivering pharmaceutical care, with responsibilities expanding beyond medication dispensing to include direct patient-centered services.^2^^,^^3^ The pharmacy workforce, particularly in developed countries, continues to experience high turnover rates and challenges with staff retention.^4^, ^5^, ^6^ The International Pharmaceutical Federation (FIP) Global Pharmacy Workforce Report identifies high turnover of skilled pharmacists as a major concern in the pharmacy setting.^7^ High turnover rates can adversely affect organizational performance, reduce workplace productivity, and compromise customer satisfaction.^8^, ^9^, ^10^ High rates of intention to leave the organization among pharmacists can also compromise the quality of pharmaceutical care services and patient safety.^8^^,^^9^ Pharmacists' turnover intention refers to their expressed intent to leave their current employer or organization in pursuit of a different position within the pharmacy field.^11^^,^^12^ Turnover intention has consistently been identified in the literature as a strong predictor of actual employee turnover and is positively associated with the likelihood of leaving a job.^13^^,^^14^

Pharmacists work across diverse practice environments, including hospitals, community, industry, and academia. Each setting presents unique job characteristics and influences the motivational factors associated with retention or departure.^15^^,^^16^ Therefore, identifying and understanding the key drivers of turnover intention is essential for developing targeted strategies to reduce pharmacist turnover and support workforce stability.

**Fig. 1:**
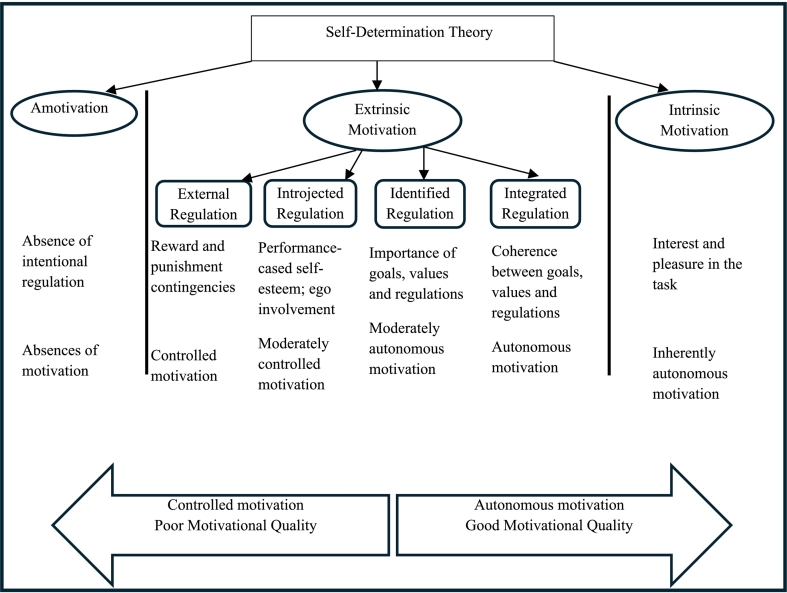
The Self-Determination Theory adapted from Ryan and Deci [22].

Organizations worldwide are increasingly assessing employee engagement levels due to its positive impact on productivity, profitability, and employee retention and loyalty.^17^ Work engagement is a positive and fulfilling work-related mindset marked by three key components: vigor (high energy and persistence), dedication (enthusiasm and sense of purpose), and absorption (deep focus and immersion in work).^18^^,^^19^ It was found that pharmacist engagement had a negative relationship with turnover intention in the community pharmacy setting.^20^ For several decades, Self-Determination Theory (SDT) has served as a foundational framework for understanding the relationship between motivation, performance, and well-being within work settings. The theory emphasizes the importance of fostering conditions that promote high-quality, sustainable motivation and voluntary engagement among both employees and customers.^21^ The distinction between autonomous and controlled motivation is central to SDT. Autonomous motivation refers to engaging in activities with a sense of volition and personal endorsement and includes both intrinsic motivation (driven by inherent interest or enjoyment) and well-internalized forms of extrinsic motivation, such as identified and integrated regulation.^21^ In contrast, controlled motivation involves external or internal pressures to act, encompassing external regulation (e.g., rewards or punishments) and introjected regulation (e.g., guilt or obligation) Fig. 1.^21^ These motivational types are arranged along a continuum of autonomy, with autonomous forms consistently linked to better performance, engagement, and well-being.^21^ In the workplace, environments that support individuals' basic psychological needs for autonomy, competence, and relatedness are more likely to foster autonomous motivation, which in turn contributes to sustainable engagement.^22^ Autonomous motivation (AM) has been linked to enhanced job performance, improved well-being, and increased resilience among healthcare professionals.^22^^,^^23^ Conversely, controlled motivation (CM) is considered a less favorable form of motivation and has been associated with higher risks of burnout.^24^^,^^25^

Kuwait is a small country located on the Arabian Peninsula, with a population of approximately 4.2 million people, the majority of whom reside in urbanized areas.^26^ The public healthcare system is structured into three levels: primary, secondary, and tertiary care. Primary care is delivered through a network of general and specialized polyclinics across five health regions, while secondary care is provided by six general hospitals.^27^ Tertiary care is offered through more than fifteen specialized medical centers distributed across the country.^27^ The implementation of clinical pharmacy services in practice remains limited to general hospitals and few private hospitals.^26^ Given the evolving role of pharmacists in Kuwait and the structural differences between public and private healthcare systems, it is essential to explore how similar factors may influence motivation, work engagement and retention in the local context.

No previous studies that have elaborated on the relationships between different types of motivation, work engagement and intention to leave the job among pharmacists have been identified. Investigating these relationships could guide organizations to develop an evidence-based retention policy for pharmacists and would enhance the supportive environment of the workplace for pharmacists to develop and deliver patient care. The objective of the present study was to identify the variables related to motivational needs and work engagement which are associated with pharmacists' intention to leave their jobs.

## 2 Methods

### 2.1 Study design and pharmacists population

This study adopted a descriptive, cross-sectional design and was conducted from October 2023 till January 2024 among clinical and nonclinical pharmacists in hospital settings in Kuwait. Only pharmacists who provided written informed consent were included in the study. Ethical approval was obtained from the Standing Committee for Coordination of Health and Medical Research at the Ministry of Health (MOH), as well as the Health Sciences Center (HSC) Ethics Committee for Student Research, prior to the initiation of any study procedures.

### 2.2 Sampling strategy, sample size and data collection procedure

A stratified purposive sampling strategy was employed to recruit pharmacists working in hospital settings across Kuwait. The sampling frame included eight major hospitals: all public general hospitals and one major private hospital (Kuwait Oil Company [KOC] Hospital). Hospitals were selected as the sole recruitment sites because, in the Kuwaiti healthcare system, clinical pharmacists are exclusively employed in hospital settings. Community pharmacies and other sectors do not currently employ clinical pharmacists in structured clinical roles, making hospitals the only appropriate and relevant context for accessing this group. The required sample size was calculated using the Raosoft sample size calculator, with the parameters set at a 95 % confidence level, 5 % margin of error, and 50 % response distribution. The recommended minimum sample size required was 280 pharmacists. To account for an anticipated 20 % non-response rate, the final target sample was increased to 336 pharmacists. This approach ensured both diversity and proportionality in the sample, enhancing the representativeness of pharmacists across different hospital settings and job functions in Kuwait. One of the researchers first contacted the pharmacy manager at each participating hospital to explain the purpose of the study and request their assistance in distributing the self-administered questionnaires to eligible pharmacists. Upon obtaining agreement, the pharmacy manager was provided with a set of printed questionnaires, along with information sheets and consent forms and was tasked with the initial distribution to pharmacists within their department. Shortly thereafter, a follow-up communication was made with the chief pharmacist at each hospital to remind them of the ongoing data collection and to encourage timely completion and return of the questionnaires. At the end of the survey period, the completed questionnaires were collected in person by the researcher from the chief pharmacists at each site.

### 2.3 Study tool

Pharmacists were provided with a self-administered, five-page questionnaire available in English or Arabic. The questionnaire consisted of 44 questions divided into four sections, which assessed work motivation (19 questions), job engagement (8 questions), intention to leave (5 questions), and demographics (12 questions). A pilot study was conducted among a sample of 15 pharmacists to evaluate the instrument's content validity, face validity, as well as the feasibility of data collection methods. The pilot participants were invited to fill out a paper-based survey in English and Arabic, and to comment on its validity and feasibility. Minor adjustments were made to the formatting and wording of certain words in order to enhance clarity, while preserving their original meaning. The pilot study data were excluded from the final analysis of the study findings.

#### 2.3.1 Multidimensional Work Motivation Scale (MWMS)

The questionnaire used in this study was the Multidimensional Work Motivation Scale (MWMS), a validated instrument developed by Gagné et al. (2015) to comprehensively assess different types of work motivation based on the Self-Determination Theory (SDT).^28^ Moreover, it has been translated and validated for use in Arabic language. The MWMS consists of 19 items measuring five core types of motivational regulation: amotivation, external regulation, introjected regulation, identified regulation, and intrinsic motivation. To capture the nuanced differences within controlled forms of motivation, the scale distinguishes between material and social external regulation. Responses to the MWMS items were rated on a 7-point Likert scale ranging from 1 (“not at all”) to 7 (“completely”).

#### 2.3.2 Utrecht Work Engagement Scale- Short Version (UWES-9)

Work engagement was assessed using the Utrecht Work Engagement Scale – Short Version (UWES-9), a validated and widely used self-report instrument developed by Schaufeli and colleagues (2006).^29^ The UWES conceptualizes work engagement as a positive, fulfilling work-related state of mind, comprising three core dimensions: vigor, dedication, and absorption. Vigor reflects high levels of energy and mental resilience while performing work tasks.

Dedication involves a strong sense of significance, enthusiasm, and pride in one's job.

Absorption refers to being fully concentrated and deeply engrossed in work activities. The UWES-9 contains nine items, with three items for each subscale, rated on a 7-point Likert scale ranging from 0 (“never”) to 6 (“always”).

#### 2.3.3 Intention to leave measure

Intent to leave was measured using a validated 5-item scale developed by Lichtenstein et al. (2004), which assesses an employee's likelihood of voluntarily exiting their current organization.^30^ The scale has demonstrated high internal consistency in prior research (Cronbach's α = 0.90). This scale has been widely used to predict actual turnover behavior and is considered a robust indicator of voluntary turnover intentions in organizational research. Responses were rated on a 7-point Likert scale ranging from 1 (strongly disagree) to 7 (strongly agree), with higher scores indicating greater intention to leave.

### 2.4 Statistical data analysis

Data management and descriptive analyses were conducted using SPSS version 28. The main method of data analysis employed in this study was structural equation modeling (SEM), with AMOS software being utilized for this purpose. The use of structural equation modeling (SEM) facilitated the simultaneous examination of the relationships between a latent multidimensional construct of work motivations (which comprises multiple measures) and other measured constructs, such as background variables. In addition to its ability to understand complex relationship structures, structural equation modeling (SEM) offered several notable advantages. For one, it accounts for measurement error, thereby providing more accurate estimates of relationships among variables than traditional analytical methods. Furthermore, structural equation modeling (SEM)’s confirmatory nature allows for the testing and comparison of theoretical models, contributing to the robustness of findings and advancing theoretical development in the field. This method's flexibility in handling both observed and unobserved variables also makes it particularly valuable for multifaceted constructs such as work motivation.

A preliminary screening of all scales was conducted before exploring different structural models among latent constructs. The assessment of internal consistency was conducted through the utilization of Cronbach's alpha reliability coefficient, followed by exploratory factor analysis (EFA) employing the principal component method. The factors derived from the exploratory factor analysis (EFA) were subsequently utilized in the confirmatory factor analysis (CFA) to establish the measurement model. After several iterations on the CFA, a measurement model was finalized and served as the foundation for structural equation modeling (SEM) to examine the hypothesized relationships among the different variables.

For the CFAs and SEMs analyses, a chi-squared statistic with degrees of freedom, and other goodness-of-fit measures such as the root mean square error of approximation (RMSEA), the comparative fit index (CFI), and the standardized root mean square residual (SRMR) were reported. A model was considered to have an acceptable fit if the comparative fit index (CFI) exceeded 0.95, the root mean square error of approximation (RMSEA) was below 0.06, and the standardized root mean square residual (SRMR) was less than or equal to 0.8. The final measurements model exhibited a chi-squared value of 1.568 (*n* = 548; *p* < 0.001), indicating a significant fit. Furthermore, the model demonstrated excellent goodness-of-fit values across all measures, including a comparative fit index (CFI) of 0.964, a root mean aquare error of approximation (RMSEA) of 0.044, and a standardized root mean square residual (SRMR) of 0.055.

### 2.5 Screening of scales

Cronbach's alpha reliability estimates for the three scales utilized in the study are displayed in Table 1. All three scales exhibited satisfactory reliability, with alpha coefficients exceeding 0.7. To ascertain the dimensionality of the scales, all items underwent principal component exploratory factor analysis (EFA) with varimax rotation. Exploratory factor analysis (EFA) with principal component extraction and varimax rotation was used to explore the structure of the Multidimensional Work Motivation Scale (MWMS). The analysis yielded a four-factor solution that aligned well with Self-Determination Theory (SDT). These factors were interpreted and labeled as follows: (1) Identified and Introjected Regulation, (2) External Regulation (social and material combined), (3) Intrinsic Motivation, and (4) Amotivation. Items were retained based on theoretical coherence and loading values, with most items exceeding the 0.70 threshold. However, two items under Identified Regulation had loadings of 0.628 and 0.675; they were retained due to their conceptual importance in the SDT framework. Each factor showed satisfactory internal consistency. (See Table 2.)

**Table 1:** Descriptive statistics for the scales used, alpha reliability coefficient and Cronbach's alpha when items were deleted.

**Scales and Items**	**Mean**	**SD**	**Corrected item-total correlation**	**Cronbach's alpha if item deleted**
**The Multidimensional Work Motivation Scale (MWMS) (Cronbach's alpha** **=** **0.783)**	**71.94**	**15.109**		
Am1:I don't, because I really feel that I'm wasting my time at work.	1.34	0.903	−0.189	0.833
Am2:I do little because I don't think this work is worth putting efforts into.	1.36	1.018	−0.268	0.836
Am3:I don't know why I'm doing this job, it's pointless work.	1.28	0.822	−0.202	0.832
Ext-Soc1:To get others' approval (e.g., supervisor, colleagues, family, clients …).	2.06	1.614	0.316	0.818
Ext-Soc2:Because others will respect me more (e.g., supervisor, colleagues, family, clients …).	2.4	1.854	0.415	0.813
Ext-Soc3:To avoid being criticized by others (e.g., supervisor, colleagues, family, clients …).	1.96	1.577	0.345	0.817
Ext-Mat1:Because others will reward me financially only if I put enough effort in my job (e.g., employer, supervisor …).	2.09	1.765	0.326	0.818
Ext-Mat2:Because others offer me greater job security if I put enough effort in my job (e.g., employer, supervisor …).	2.32	1.818	0.488	0.809
Ext-Mat3:Because I risk losing my job if I don't put enough effort in it.	2.1	1.669	0.398	0.814
Introj1:Because I have to prove to myself that I can.	5.21	1.869	0.591	0.802
Introj2:Because it makes me feel proud of myself.	5.83	1.61	0.661	0.8
Introj3:Because otherwise I will feel ashamed of myself.	4.99	2.136	0.612	0.8
Introj4:Because otherwise I will feel bad about myself.	5.35	1.974	0.616	0.8
Ident1:Because I personally consider it important to put efforts in this job.	6.1	1.4	0.427	0.813
Ident2:Because putting efforts in this job aligns with my personal values.	6.15	1.385	0.461	0.811
Ident3:Because putting efforts in this job has personal significance to me.	5.86	1.463	0.563	0.806
Intrin1:Because I have fun doing my job.	5.38	1.802	0.486	0.809
Intrin2:Because what I do in my work is exciting.	5.01	1.826	0.463	0.81
Intrin3:Because the work I do is interesting.	5.13	1.765	0.458	0.811
**Utrecht Work Engagement Scale-9 (UWES-9) (Cronbach's alpha** **=** **0.954)**	**39.31**	**11.988**		
VI1:At my work, I feel bursting with energy.	4.21	1.501	0.868	0.946
VI2:At my job, I feel strong and vigorous.	4.29	1.558	0.873	0.945
DE2:I am enthusiastic about my job.	4.38	1.5	0.904	0.944
DE3:My job inspires me.	4.15	1.639	0.829	0.947
VI3:When I get up in the morning, I feel like going to work.	3.76	1.872	0.72	0.955
AB3:I feel happy when I am working intensely	4.19	1.674	0.786	0.95
DE4:I am proud on the work that I do.	5.15	1.25	0.728	0.953
AB4:I am immersed in my work.	4.67	1.442	0.79	0.949
AB5:I get carried away when I'm working.	4.51	1.521	0.873	0.945
**Intention to leave (Cronbach's alpha** **=** **0.919)**	**16.87**	**8.625**		
There is a good chance that I will leave the Ministry of Health (or my current organization) in the next year.	2.96	1.955	0.742	0.91
There is a good chance that I will leave the Ministry of Health (or my current organization) in the next 5 years.	3.86	1.971	0.787	0.901
I frequently think of leaving the Ministry of Health (or my current organization).	3.46	2.073	0.836	0.891
I will probably look for a new organization in the next year.	2.89	1.86	0.796	0.9
I will probably look for a new organization in the next 5 years.	3.71	2.06	0.796	0.9

**Abbreviation:** Am, Amotivation; Ext-Soc, External-Social; Ext-Mat, External-Material; Introj, Introjected; Ident, Identified; Intrin, Intrinsic. VI, Vigor; DE; Dedication, AA, Absorption.

**Table 2:** Standardized item loadings from an exploratory factor analysis (using principal components extraction) revealed a four-factor solution for the Multidimensional Work Motivation Scale (MWMS).

**Items**	**Factors**			
	1	2	3	**4**
Am1:I don't, because I really feel that I'm wasting my time at work.				0.849
Am2:I do little because I don't think this work is worth putting efforts into.				0.786
Am3:I don't know why I'm doing this job, it's pointless work.				0.872
Ext-Soc1:To get others' approval (e.g., supervisor, colleagues, family, clients …).		0.714		
Ext-Soc2:Because others will respect me more (e.g., supervisor, colleagues, family, clients …).		0.774		
Et-Soc3:To avoid being criticized by others (e.g., supervisor, colleagues, family, clients …).		0.784		
Ext-Mat1:Because others will reward me financially only if I put enough effort in my job (e.g., employer, supervisor …).		0.821		
Ext-Mat2:Because others offer me greater job security if I put enough effort in my job (e.g., employer, supervisor …).		0.836		
Ext-Mat3:Because I risk losing my job if I don't put enough effort in it.		0.762		
Introj1:Because I have to prove to myself that I can.	0.725			
Introj2:Because it makes me feel proud of myself.	0.775			
Introj3:Because otherwise I will feel ashamed of myself.	0.796			
Introj4:Because otherwise I will feel bad about myself.	0.827			
Ident1:Because I personally consider it important to put efforts in this job.	0.717			
Ident2:Because putting efforts in this job aligns with my personal values.	0.628			
Ident3:Because putting efforts in this job has personal significance to me.	0.675			
Intrin1:Because I have fun doing my job.			0.820	
Intrin2:Because what I do in my work is exciting.			0.879	
Intrin3:Because the work I do is interesting.			0.898	

**Notes:** Factor 1: Identified and Introjected Regulation, Factor 2: External Regulation (Social and Material), Factor 3: Intrinsic Motivation, Factor 4: Amotivation.

**Abbreviation:** Am, Amotivation; Ext-Soc, External-Social; Ext-Mat, External-Material; Introj, Introjected; Ident, Identified; Intrin, Intrinsic;

Table 3, Table 4 present single-factor solutions for Work Engagement and Intention to Leave, respectively, each demonstrating good reliability and item loadings.

**Table 3:** Standardized item loadings from an exploratory factor analysis (using principal components extraction) revealed a one-factor solution for the Job Engagement Scale.

**Items**	**Factor** **(Job Engagement)**
VI1:At my work, I feel bursting with energy.	0.897
VI2:At my job, I feel strong and vigorous.	0.905
VI3:When I get up in the morning, I feel like going to work.	0.771
DE2:I am enthusiastic about my job.	0.928
DE3:My job inspires me.	0.867
DE4:I am proud on the work that I do.	0.784
AB3:I feel happy when I am working intensely.	0.831
AB4:I am immersed in my work.	0.839
AB5:I get carried away when I'm working.	0.906

**Abbreviation:** VI, Vigor; DE; Dedication, AA, Absorption.

**Table 4:** Standardized item loadings from an exploratory factor analysis (using principal components extraction) revealed a one-factor solution for the Intention to Leave Scale.

**Items**	**Factor** **(Intention to Leave)**
There is a good chance that I will leave the Ministry of Health (or my current organization) in the next year.	0.835
There is a good chance that I will leave the Ministry of Health (or my current organization) in the next 5 years.	0.864
I frequently think of leaving the Ministry of Health (or my current organization).	0.901
I will probably look for a new organization in the next year.	0.872
I will probably look for a new organization in the next 5 years.	0.874

### 2.6 Structural model

Structural equation modeling (SEM) was developed to examine the relationship between the multidimensional work motivation, job engagement and intention to leave, all were first-order latent constructs, while also considering the influence of background demographic, as directly measured constructs.

## 3 Results

A total of 336 questionnaires were distributed to pharmacists, out of which 288 were collected, resulting in a response rate of 85.7 %. Demographic information is presented in Table 5. The majority of participating pharmacists were female (71.2 %), and most fell within the 23–39-year age range (76.7 %). Furthermore, the majority were of Kuwaiti nationality (69.5 %). Regarding educational background, 67 % held a bachelor's degree, while 14.6 % had earned a Doctor of Pharmacy (PharmD) degree. In terms of professional experience, 46 % (*n* = 127) reported having worked in the field for 1 to 5 years. Engagement in clinical pharmacy practice was limited, with 73.5 % of respondents indicating non-involvement.

**Table 5:** Demographic characteristics (*N* = 288).

**Characteristics**	**n**	**%**
**Gender**		
Male	83	28.8
Female	205	71.2
**Age, years (Mean** **=** **33.27)**	**n**	**%**
23–39	203	76.6
40–49	49	18.5
50–59	10	3.8
60 or more	3	1.1
Missing	23	
**Nationality**	**n**	**%**
Kuwaiti	200	69.5
Non-Kuwaiti	86	30.1
Missing	2	0.7
**Highest Professional Degree**	**n**	**%**
BSc.	193	67
PharmD	42	14.6
Clinical post grad degree (Msc/PhD)	37	12.8
Other post grad degree (Msc/PhD)	16	5.6
**Country of first pharmacy degree**	**n**	**%**
Kuwait	116	41.1
Middle east countries	116	41.1
Westernized countries	50	17.8
Missing	6	2.1
**Country of post graduate degree**	**n**	**%**
Kuwait	29	32.6
Middle East Countries	12	13.5
Westernized Countries	48	53.9
**Employment**	**n**	**%**
Manager	4	1.4
Staff	284	98.6
**Hospitals**	**n**	**%**
Al-Amiri	30	10.4
Mubarak Al-Kabeer	53	18.4
Al-Farwaniya	53	18.4
Al-Adan	31	10.8
Al-Jahra	28	9.7
Al-Sabah	25	8.7
Jaber	29	10.1
KOC	39	13.5
**How long have you worked as a pharmacist inside Kuwait? (Mean** **=** **7.72)**	**n**	**%**
Less than 1 year	16	5.9
1 year - 5 years	127	46
6 years - 10 years	46	16.7
11 years - 15 years	45	16.3
16 years - 20 years	27	9.8
>20 years	14	5.2
Missing	13	4.5
**Clinical Pharmacy** **Practice**	**n**	**%**
Yes	75	26.5
No	208	73.5
Missing	5	
Percentage of time dedicated to clinical services **(Mean** **= 77.59 %, SD** **=** **28.35, Median** **=** **100, Mode** **=** **100)**	**n**	**%**
0 %	1	1.3
33 %	12	16
40 %	5	6.7
50 %	2	2.7
60 %	3	4
70 %	2	2.7
75 %	1	1.3
80 %	6	8
83 %	1	1.3
90 %	3	4
96 %	1	1.3
100 %	38	50.7

### 3.1 Factors associated with the intention to leave

**Fig. 2:**
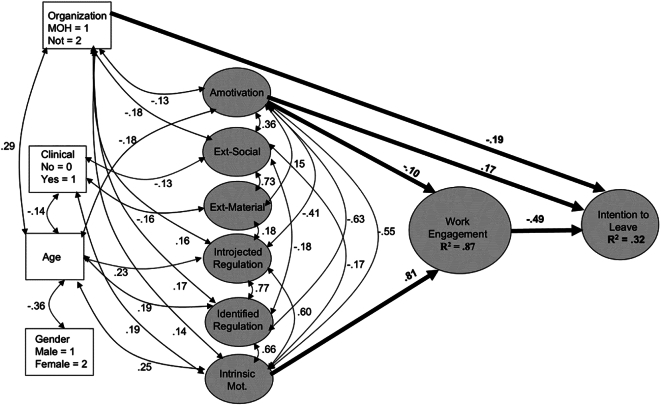
Structural equation model of Intention to Leave as a function of Engagement, Motivation, and background variables χ^2^ (…, *n* = 548) = 1.568 (*p* < 0.001), CFI = 0.964, RMSEA = 0.044, SRMR = 0.055. All paths are statistically significant at *p* < 0.05. Paths representing effects of background variables are drawn in thinner lines.

The findings of the SEM analysis examining the association between work engagement and intention to leave are presented in Fig. 2. The model was evaluated using various indices, which yielded a chi-square value of 1.568 (*n* = 548; *p* = 0.001). The goodness-of-fit measures indicated an excellent fit across all indicators, with a comparative fit index (CFI) of 0.964 and a root mean square error of approximation (RMSEA) of 0.044. The path coefficients depicted in Fig. 2 were found to be statistically significant at *p* < 0.05. The obtained standard regression coefficient (β = − 0.49) suggests that work engagement has a statistically significant negative effect on the intention to leave. It was found that amotivation has a statistically significant positive impact on the intention to leave, as indicated by a standard regression coefficient (beta) of 0.17. Additionally, amotivation was found to have a negative impact on work engagement, with a beta coefficient of −0.10. The obtained standard regression coefficient (β = 0.81) suggests that intrinsic motivation has a statistically significant and positive effect on work engagement. Working in public hospitals, as compared to a private hospital, was found to have a statistically significant negative effect on the intention to leave, with a standardized regression coefficient (β) of −0.19. In addition, organization type was also found to be associated with motivation: amotivation: (beta = − 0.13), extrinsic social motivation (beta = − 0.18), introjected regulation (beta = 0.16), identified regulation (beta = 0.17), and intrinsic motivation (beta = 0.14). Additionally, it was found that the relatively younger age of pharmacists was correlated with their intention to leave the Ministry of Health. Age was also found to be significantly associated with various types of motivation, including amotivation (β = − 0.18), introjected regulation (β = 0.23), identified regulation (β = 0.19), and intrinsic motivation (β = 0.25). In addition, there was a correlation between participants' age and their type of practice (− 0.14), and gender (− 0.36). The practice of clinical pharmacy was found to be linked to both extrinsic motivation, specifically social (beta = −0.13) and material (beta = −0.16), as well as intrinsic motivation (beta = 0.25).

### 3.2 Effect of hospital sites on intension to leave and amotivation

A one-way between-groups analysis of variance was conducted to explore the impact of different hospital sites on the employees' intention to leave. A statistically significant difference in intention-to-leave scores was observed across the eight hospitals (F(7, 279) = 6.2, *p* < 0.001). The difference in mean scores between the groups was large. The effect size calculated using eta squared was 0.135. Post-hoc comparisons using the Tukey HSD test indicated that the mean score for KOC hospital (*M* = 10.08, *SD* = 6.78) was significantly different from Amiri hospital (*M* = 16.83, *SD* = 7.4), Mubarak Al-Kabeer hospital (*M* = 16.56, *SD* = 8.85), Farwaniya hospital (*M* = 20, *SD* = 8.48), Adan hospital (*M* = 17.84, *SD* = 9.53), Jahra hospital (*M* = 16.43, *SD* = 7.95) and Jaber hospital (*M* = 20.9, *SD* = 7.23).

A one-way between-groups analysis of variance was also conducted to explore the impact of different hospital sites on the amotivation score. There was a statistically significant difference at the *p* < 0.05 in the amotivation score across the eight hospitals: F (7, 279) = 3.94, *p* ≤0.001. The actual difference in mean scores between the groups was medium. The effect size calculated using eta squared was 0.09. Post-hoc comparisons using the Tukey HSD test indicated that the mean score for Sabah hospital (*M* = 5.68, *SD* = 5.31) was significantly different from Mubarak Al-Kabeer hospital (*M* = 3.58, *SD* = 1.622), Adan hospital (*M* = 3.55, *SD* = 1.98), Jahra hospital (*M* = 3.36, *SD* = 0.99) and KOC hospital (*M* = 3.18, *SD* = 0.644). In addition, the mean score for Jaber hospital (*M* = 5.03, *SD* = 2.73) was significantly different from KOC hospital (M = 3.18, SD = 0.644).

## 4 Discussion

This study explored the relationships between different types of motivation, work engagement, and pharmacists' intention to leave, using Self-Determination Theory as a guiding framework. The findings showed that autonomous motivation—such as identified regulation and intrinsic motivation—was strongly associated with higher work engagement, while amotivation and external material regulation were linked to lower engagement and greater intention to leave. These results highlight the importance of fostering meaningful and internally driven motivation among pharmacists to enhance their engagement and reduce turnover intentions.

The model explained a substantial portion of the variance in work engagement (87 %) and a moderate portion in intention to leave (32 %). This means that nearly one-third of the differences in pharmacists' intention to leave their jobs in Kuwait can be statistically explained by the motivational factors, engagement levels, and organizational context (public vs. private) included in the model. While this indicates that these are important influences, it also suggests that other unmeasured factors may contribute to turnover intentions and warrant further investigation.

Pharmacists working in public hospitals had higher turnover intention than those in private hospitals. In addition, there were significant differences in pharmacists' intention to leave across different public hospital settings. A systematic review examining pharmacists' turnover and turnover intention found that the average score for community pharmacists' intention to leave their job was significantly lower than that of hospital pharmacists' turnover intention intensity in Asian countries.^31^ However, the current study was conducted only at public and private hospital settings in Kuwait. The distinction between private and public hospital settings can be influenced by various factors, including promotion and advancement opportunities, payment, organizational resources, and policies.^32^^,^^33^ Additionally, the differences in pharmacists' intention to leave in different public hospital settings can be influenced by organizational support and organizational climate.^32^ Different job setting (public vs. private) also predicted various types of motivation. For example, public hospital pharmacists reported lower amotivation and higher intrinsic and identified motivation. These types of autonomous motivation has been linked with lower turnover intentions among health care professionals.^23,^^34^ In contrast, another study found no significant association between the work motivation of clinical pharmacists practicing in either private or public healthcare sector hospitals.^35^ Similarly, the type of hospital did not have an influence on work motivation of medical specialists.^23^

The results of this study showed that low motivation is a key factor influencing both pharmacists' intention to leave their jobs and their level of work engagement. However, no previous studies have examined this relationship through the lens of Self-Determination Theory (SDT). The findings of the current study revealed that the practice of clinical pharmacy was positively associated with intrinsic motivation, while being negatively associated with both social and material extrinsic motivations. This suggests that pharmacists in clinical roles, as observed in this study, are more internally driven by interest and professional fulfillment rather than by external rewards or recognition. A study conducted in South Africa among clinical pharmacists found higher levels of amotivation among graduates who were not practicing as clinical pharmacists compared to those employed in clinical roles.^35^ Additionally, the same study reported that graduates who did not receive extra financial compensation for providing clinical services also exhibited greater levels of amotivation.^35^

Furthermore, this study found that pharmacists less than 40 years old working in public hospital settings were more likely to express an intention to leave, which appears to be linked to their type of motivation. Specifically, pharmacists less than 40 years old exhibited lower levels of autonomous motivation, while older pharmacists demonstrated higher levels of intrinsic motivation and were more responsive to internalized regulatory mechanisms, potentially contributing to their greater engagement and lower turnover intention. This contrasts with the findings of Crafford et al. (2021),^35^ who reported no significant association between age and work motivation among clinical pharmacists. Additionally, another study found that specialists younger than 50 years scored higher on intrinsic motivation and self-regulation.^23^ The study conducted by Crafford et al. (2021) revealed that graduate pharmacists who were not practicing as clinical pharmacists exhibited higher levels of amotivation compared to those employed in a dedicated clinical position.^35^ The current results revealed that clinical pharmacists reported significantly lower levels of external social and external material motivation compared to non-clinical pharmacists. This suggests that those working in clinical roles are less driven by external pressures or tangible rewards and may instead be more internally motivated. These findings align with the nature of clinical pharmacy practice, which often provides more opportunities for patient-centered engagement and professional fulfillment.

## 5 Strengths and limitations

One of the strengths of this study was that it capitalized on the methodological advantages of structural equational modeling (SEM) that handle complex relationships among multiple variables simultaneously. Structural equation modeling (SEM) allows for the examination of both direct and indirect effects in a comprehensive model, providing a more nuanced understanding of relationships that may not be detectable using regression analysis. Additionally, SEM incorporates measurement error, which results in a more accurate representation of constructs. It distinguishes between observed variables and their underlying latent constructs, resulting in more reliable and valid findings. Intention to leave and work engagement were conceptualized as latent constructs formed by combining the measured variables. SEM allowed for the estimation of the relationship between work engagement and intention to leave, while simultaneously controlling for the effect of multiple background variables. However, this study has several limitations that should be acknowledged. First, the use of a cross-sectional design limits the ability to draw causal inferences between job characteristics, motivation, engagement, and intention to leave. The associations identified represent correlations at a single point in time and do not account for changes over time or the directionality of these relationships. Second, the potential for non-response bias may have influenced the results, as individuals with strong opinions or particular experiences may have been more likely to participate, potentially limiting the representativeness of the findings. Third, the study was conducted exclusively in Kuwait, and while it provides valuable insights into the local context, the generalizability of the results to other countries or healthcare systems, particularly those with different organizational structures or cultural dynamics, may be limited. Future studies using longitudinal or mixed-method designs and including diverse settings are recommended to validate and extend these findings.

## 6 Implications for practice

Given the observed differences in motivation between pharmacists in clinical and non-clinical roles, and between those in public versus private sectors in Kuwait, further research is needed to explore the specific workplace factors influencing motivation in these settings. Future studies should investigate how organizational culture, leadership style, recognition, and job autonomy may vary across hospital types and how they shape pharmacists' motivation. Additionally, motivation and engagement should be regularly assessed to identify at-risk groups, with interventions tailored by age group to address differing motivational drivers.

## 7 Conclusion

This study provides key evidence that connects pharmacists' work motivation and engagement to their decisions to leave their job in Kuwait. The findings indicate that intrinsic motivation and work engagement decrease turnover intention, while amotivation increases the likelihood of pharmacists leaving their job. Furthermore, the organizational setting, particularly the difference between public and private hospitals, significantly influences motivation and retention. Younger pharmacists and those with lower engagement are more inclined to leave, highlighting the importance of retention strategies tailored to age and context.

## Financial disclosures and conflicts of interest

The authors declares no financial disclosures or conflicts of interest related to this study.

## CRediT authorship contribution statement

**Muna Sabah Murad:** Writing – review & editing, Writing – original draft, Software, Project administration, Investigation, Formal analysis, Data curation, Conceptualization. **Mohammad Waheedi:** Writing – review & editing, Methodology, Investigation, Formal analysis, Data curation, Conceptualization. **Fatima Jeragh Alhaddad:** Writing – review & editing. **Maryam Yousef Baqer:** Writing – review & editing. **Farah Atallah Alenezi:** Writing – review & editing.

## Declaration of competing interest

The authors declare that they have no known competing financial interests or personal relationships that could have appeared to influence the work reported in this paper.

## Appendix A Supplementary data

Supplementary material Image 1
