# Supplementary material for: Driven to stay or leave: Exploring motivation, engagement, and turnover intentions among pharmacists in the healthcare system
Source: Explor Res Clin Soc Pharm. 2025 Aug 22;20:100645. doi: 10.1016/j.rcsop.2025.100645 (PMC12410516; doi:10.1016/j.rcsop.2025.100645)
Supplement: Supplementary file 1 — Supplementary material [file mmc1.docx]

**Clinical Pharmacy Practice: motivation, work-related outcomes after formal Implementation**

**Section 1: Information about yourself**

1. **Gender:** 🗆 Male 🗆 Female
2. **Age (in years):** …………….…
3. **Nationality:** 🗆 Kuwaiti 🗆 Non-Kuwaiti (…………..……………..please specify)
4. **Degree: (Select all that apply)**

🗆 BSc.

🗆 PharmD

🗆 Clinical Post grad degree (MSc/PhD)

🗆 Other Post grad degree (MSc/PhD)

1. **Country where first pharmacy degree was obtained**………………………………………………….
2. **Country where post graduate degree(s) was obtained**……………………………………………….
3. **How long have you worked as a pharmacist?**

🗆 Inside Kuwait …………. years 🗆 Outside Kuwait ………..……. years

1. **Employer or employee status:** 🗆 Owner or Manager 🗆 Staff Pharmacist
2. **Place of practice:** 🗆 Ministry of Health (MOH) 🗆 Private Sector 🗆 Oil Sector
3. **What is your specific place of practice?** 🗆 Hospital 🗆Polyclinic 🗆Other
4. **Health district:**

🗆 Al-Asma 🗆 Hawali 🗆 Al-Farwaniya 🗆 Al-Ahmadi 🗆 Al-Jahra

🗆 Al-Sabah 🗆 Mubarak Alkabeer

1. **Do you currently practice clinical** **pharmacy?** 🗆 Yes 🗆 No
2. **If you currently practice clinical pharmacy, what is the percentage of your time dedicated to the clinical function? ……………%**

For example,

If your total time is dedicated to clinical services, then it is 100%

If you spend 2 hrs out of 6 hrs a day, that is 33%

If you spend 2 days out of 5 workdays in a week, that is 40%

**Section 2: The following statements are possible answers to the following question:**

**“Why do you or would you put efforts into your current job?”.**

"لماذا تقوم بعملك الحالي؟"

1.I don’t because I really feel that I'm wasting my time at work.

1.أنا لا أقوم بعملي ,لأنني حقاً أشعر بأني أضيع وقتي في العمل .

Not at all Very little A little Moderately Strongly Very strongly Completely

1 2 3 4 5 6 7

2.I do little because I don’t think this work is worth putting efforts into.

2. لا أفعل شيئاً يذكر لأنني لا أعتقد أن هذا العمل يستحق أن أبذل فيه جهداً.

Not at all Very little A little Moderately Strongly Very strongly Completely

1 2 3 4 5 6 7

3.I don’t know why I’m doing this job, it’s pointless work.

3. أنا لا أعرف لماذا أقوم بهذا العمل, فهو عمل لا طائل منه.

Not at all Very little A little Moderately Strongly Very strongly Completely

1 2 3 4 5 6 7

4.To get others’ approval (e.g., supervisor, colleagues, family, patients, etc.).

4. للحصول على اعتماد الآخرين (على سبيا المثال, المدير, الزملاء, العائلة, المرضى).

Not at all Very little A little Moderately Strongly Very strongly Completely

1 2 3 4 5 6 7

5.Because others will respect me more (e.g., supervisor, colleagues, family, patients, etc.).

5.لأن الآخرين سيحترمونني بشكل أكبر (على سبيا المثال, المدير, الزملاء, العائلة, المرضى).

Not at all Very little A little Moderately Strongly Very strongly Completely

1 2 3 4 5 6 7

6.To avoid being criticized by others (e.g., supervisor, colleagues, family, patients, etc.).

6.لتجنب انتقاد الآخرين (على سبيا المثال, المدير, الزملاء, العائلة, المرضى).

Not at all Very little A little Moderately Strongly Very strongly Completely

1 2 3 4 5 6 7

7.Because others will reward me ﬁnancially only if I put enough effort in my job (e.g., employer, supervisor, etc.).

7.لأنني لن أحصل على راتبي إلا إذا قدمت جهداً كافياً في عملي.

Not at all Very little A little Moderately Strongly Very strongly Completely

1 2 3 4 5 6 7

8.Because others offer me greater job security if I put enough effort in my job (e.g., employer, supervisor, etc.).

8.لأن الآخرين يقدمون لي المزيد من الأمن الوظيفي, اذا قدمت جهداً كافياً في عملي (المدير).

Not at all Very little A little Moderately Strongly Very strongly Completely

1 2 3 4 5 6 7

9.Because I risk losing my job if I don’t put enough effort in it.

9.لأنني أخاطر بفقدان وظيفتي إذا لم أقم بجهد كاف فيها.

Not at all Very little A little Moderately Strongly Very strongly Completely

1 2 3 4 5 6 7

10.Because I have to prove to myself that I can.

10.لأنني يجب أن أثبت لنفسي أنني أستطيع القيام بعملي.

Not at all Very little A little Moderately Strongly Very strongly Completely

1 2 3 4 5 6 7

11.Because it makes me feel proud of myself.

11.لأنني يجعلني أشعر بالفخر بنفسي.

Not at all Very little A little Moderately Strongly Very strongly Completely

1 2 3 4 5 6 7

12.Because otherwise I will feel ashamed of myself.

12.لأنني إذا لم أقم بعملي سأشعر بالعار من نفسي.

Not at all Very little A little Moderately Strongly Very strongly Completely

1 2 3 4 5 6 7

13.Because otherwise I will feel bad about myself.

13.لأنني إذا لم أقم بعملي سوف أشعر بالضيق من نفسي.

Not at all Very little A little Moderately Strongly Very strongly Completely

1 2 3 4 5 6 7

14.Because I personally consider it important to put efforts in this job.

14.لأنني شخصياً أعتبر أنه من المهم بذل الجهود في هذه الوظيفة.

Not at all Very little A little Moderately Strongly Very strongly Completely

1 2 3 4 5 6 7

15.Because putting efforts in this job aligns with my personal values.

15.لأن القيام بهذه الوظيفة يتماثل مع قيمي الشخصية.

Not at all Very little A little Moderately Strongly Very strongly Completely

1 2 3 4 5 6 7

16.Because putting efforts in this job has personal signiﬁcance to me.

16.لأن القيام بهذه الوظيفة له أهمية شخصية.

Not at all Very little A little Moderately Strongly Very strongly Completely

1 2 3 4 5 6 7

17.Because I have fun doing my job.

17.لأنني أجد المتعة في القيام بوظيفتي.

Not at all Very little A little Moderately Strongly Very strongly Completely

1 2 3 4 5 6 7

18.Because what I do in my work is exciting.

18.لأن ما أقوم به في عملي مثير.

Not at all Very little A little Moderately Strongly Very strongly Completely

1 2 3 4 5 6 7

19.Because the work I do is interesting.

19.لأن العمل الذي أقوم به ممتع.

Not at all Very little A little Moderately Strongly Very strongly Completely

1 2 3 4 5 6 7

**Section 3: The following is a number of statements regarding about how you feel at work.**

**Read each statement carefully and** **decide how frequently you feel that way.**

1.At my work, I feel bursting with energy.

1. أشعر أنني مفعم/ة بالطاقة في العمل

Never Almost never Rarely Sometimes Often Very often Always

0 1 2 3 4 5 6

2.At my job, I feel strong and vigorous.

2. أشعر بقوة ونشاط خلال قیامي بوظیفتي.

Never Almost never Rarely Sometimes Often Very often Always

0 1 2 3 4 5 6

3.I am enthusiastic about my job.

أنا متحمس لوظیفتي..3

Never Almost never Rarely Sometimes Often Very often Always

0 1 2 3 4 5 6

4.My job inspires me.

4. وظیفتي تلھمني.

Never Almost never Rarely Sometimes Often Very often Always

0 1 2 3 4 5 6

5.When I get up in the morning, I feel like going to work.

عندما أستيقظ في الصباح أرغب في الذھاب إلى العمل. .5

Never Almost never Rarely Sometimes Often Very often Always

0 1 2 3 4 5 6

6.I feel happy when I am working intensely.

6.أشعر بالسعادة عندما أعمل بشكل مكثف.

Never Almost never Rarely Sometimes Often Very often Always

0 1 2 3 4 5 6

7.I am proud on the work that I do.

.7.أنا فخور/ة بعملي

Never Almost never Rarely Sometimes Often Very often Always

0 1 2 3 4 5 6

8.I am immersed in my work.

.أنا منغمس/ة في عملي.8

Never Almost never Rarely Sometimes Often Very often Always

0 1 2 3 4 5 6

9. I get carried away when I’m working.

9.یتملكني الحماس عندما أعمل.

Never Almost never Rarely Sometimes Often Very often Always

0 1 2 3 4 5 6

**Section 4: Intention to Leave**

**The following section is a number of statements regarding your intention to leave your organization. Read each statement carefully. Then, indicate your level of agreement with each statement**

1.There is a good chance that I will leave the Ministry of Health (or my current organization) in the next year .

Strongly disagree Disagree Somewhat disagree Uncertain Somewhat agree Agree Strongly agree

1 2 3 4 5 6 7

2.There is a good chance that I will leave the Ministry of Health (or my current organization) in the next 5 years.

Strongly disagree Disagree Somewhat disagree Uncertain Somewhat agree Agree Strongly agree

1 2 3 4 5 6 7

3.I frequently think of leaving the Ministry of Health (or my current organization).

Strongly disagree Disagree Somewhat disagree Uncertain Somewhat agree Agree Strongly agree

1 2 3 4 5 6 7

4.I will probably look for a new organization in the next year.

Strongly disagree Disagree Somewhat disagree Uncertain Somewhat agree Agree Strongly agree

1 2 3 4 5 6 7

5.I will probably look for a new organization in the next 5 years.

Strongly disagree Disagree Somewhat disagree Uncertain Somewhat agree Agree Strongly agree

1 2 3 4 5 6 7

6. If you have intention to leave, what will be the reason?

**…………………………………………………………………………………………………………………………**

**…………………………………………………………………………………………………………………………**

**…………………………………………………………………………………………………………………………**

**End of Questions**
